# Implicit measures of anthropomorphism: affective priming and recognition of apparent animal emotions

**DOI:** 10.3389/fpsyg.2023.1149444

**Published:** 2023-07-07

**Authors:** Mike Dacey, Jennifer H. Coane

**Affiliations:** ^1^Department of Philosophy, Bates College, Lewiston, ME, United States; ^2^Department of Psychology, Colby College, Waterville, ME, United States

**Keywords:** anthropomorphism, priming, emotion, aging, affective priming

## Abstract

It has long been recognized that humans tend to anthropomorphize. That is, we naturally and effortlessly interpret the behaviors of nonhuman agents in the same way we interpret human behaviors. This tendency has only recently become a subject of empirical research. Most of this work uses explicit measures. Participants are asked whether they attribute some human-like trait to a nonhuman agent on some scale. These measures, however, have two limitations. First, they do not capture automatic components of anthropomorphism. Second, they generally only track one anthropomorphic result: the attribution (or non-attribution) of a particular trait. However, anthropomorphism can affect how we interpret animal behavior in other ways as well. For example, the grin of a nonhuman primate often looks to us like a smile, but it actually signals a state more like fear or anxiety. In the present work, we tested for implicit components of anthropomorphism based on an affective priming paradigm. Previous work suggests that priming with human faces displaying emotional expressions facilitated categorization of words into congruent emotion categories. In Experiments 1–3, we primed participants with images of nonhuman animals that appear to express happy or sad emotions, and asked participants to categorize words as positive or negative. Experiment 4 used human faces as control. Overall, we found consistent priming congruency effects in accuracy but not response time. These appeared to be more robust in older adults. They also appear to emerge with more processing time, and the pattern was the same with human as with primate faces. This demonstrates a role for automatic processes of emotion recognition in anthropomorphism. It also provides a potential measure for further exploration of implicit anthropomorphism.

## Introduction

The human tendency to anthropomorphize has been a focus of increasing empirical research in the last 15 years or so. To anthropomorphize, in the broadest sense of the term, is to interpret the actions or behaviors of a nonhuman agent as if it were human. Going back to, arguably, the 6th century B.C.E., it has been common to describe anthropomorphism as a human tendency that is common and difficult to avoid, perhaps even compulsory and innate (Epley, 2018). Despite the long history of awareness of anthropomorphism, it had gone surprisingly neglected as a topic of study in psychology until relatively recently.

There are a number of different reasons that researchers have begun to pursue empirical work on anthropomorphism. Anthropomorphism of spiritual entities plays an important role in the psychology of religion (Barrett and Keil, 1996; Shaman et al., 2018). Anthropomorphism and its inverse, dehumanization, have significant influence on empathy and moral psychology (Waytz et al., 2010b; Gray et al., 2012). Researchers in comparative (animal) psychology have long worried that a human tendency to anthropomorphize might lead to errors in the field (e.g., de Waal, 1999; Sober, 2005; Dacey, 2017; Williams et al., 2020). Most recently, anthropomorphism has been of substantial interest to those in social robotics and user interface design, attempting to make machines that humans will interact with positively (Van Doorn et al., 2017; Hortensius et al., 2018; Chugunova and Sele, 2020; Sheridan, 2020; Harris and Anthis, 2021).

The majority of empirical work on the subject has used explicit reports by participants. Perhaps the best-known example is the Individual Differences in Anthropomorphism Questionnaire (IDAQ) (Waytz et al., 2010a). The IDAQ is a 30-item questionnaire that asks participants to answer questions about various entities, and whether the participants attribute to them some trait or property on an 11-point scale (from *not at all* to *very much*). Non-anthropomorphic control questions include “To what extent is the forest durable?” and “To what extent is the average robot good-looking?” Anthropomorphic target questions include “To what extent do cows have intentions?” and “To what extent does a car have free will?” These explicit measures have value. For instance, IDAQ scores are reasonably stable over time, and correlate with the practical judgments we might expect: For example, those with higher IDAQ scores are harsher moral judges of actions that destroy machines or plants, and report greater trust in machines (Waytz et al., 2010a). Questionnaire-based measures are also adaptable, as they can be paired with a number of interventions before and during work on the questionnaire, and the questions themselves can be adapted to a particular focus. This is how Epley and colleagues, for example, have built up evidence for their model of anthropomorphism, known as the “three factor” model (Epley et al., 2007): strategically manipulating each of their proposed factors, and observing the effect (e.g., Epley et al., 2008; Waytz et al., 2010c). Despite these strengths, explicit measures of anthropomorphism have two key limitations.

First, as with explicit measures generally, they cannot directly probe the contributions that unconscious processing makes to anthropomorphism (see Jacoby, 1991). Anthropomorphism is generally seen as a compulsory tendency, suggesting that it has important unconscious components. There is some more direct evidence for this as well. Nass and Moon (2000; see also Nass et al., 1993) found that participants treated a computer as a social agent even when they were unwilling to explicitly ascribe anthropomorphic traits. Złotowski et al. (2018) argue that this is a case of a dissociation between “Type 1” automatic processes (driving the more natural interactions) and “Type 2” controlled processes (driving the explicit reports). We follow Złotowski et al. (2018) by calling these, in turn, *implicit* and *explicit* anthropomorphism.

Złotowski et al. (2018) argue that implicit anthropomorphism has been neglected. They tested their “dual-process” model by attempting to independently manipulate implicit and explicit anthropomorphic responses. Participants interacted with a robot named Robovie in a game. Afterward, they were asked to explicitly report anthropomorphic trait attributions to Robovie. The implicit measure was a priming paradigm: Participants were primed with an image of either Robovie or another robot before categorizing a black-and-white silhouette as either a human or object. The prediction was that implicit anthropomorphism of Robovie would facilitate categorization of human silhouettes. To manipulate explicit anthropomorphism, they presented prompts to manipulate motivation on the task. To manipulate implicit anthropomorphism, they manipulated the apparent emotionality of Robovie in the game.

The authors did find the predicted priming effect in their implicit measure. However, they were unable to manipulate the implicit and explicit measures independently. They attributed this to complicated interrelations between psychological processes, but also note that the validity of the implicit measure could be questioned: There are no validated measures of implicit anthropomorphism against which it could be tested. There is, thus, a need for further development of measures of implicit anthropomorphism, as highlighted by Złotowski et al. (2018).

Work on social robotics and human-machine interaction has recently introduced some other intriguing candidates. These often track emotional engagement with machines during human-machine interactions, using galvanic skin response, electrocardiogram, and facial expressions of participants (e.g., Rosenthal-von der Pütten et al., 2013; Waytz et al., 2014; Menne and Lugrin, 2017), or even fMRI of emotional centers of the brain (Cross et al., 2019). Other studies have used eye tracking. Staudte and Crocker (2009) tracked participants’ gaze to see if they followed a robot’s “gaze” when attempting to understand its speech. Thellman et al. (2020) used gaze tracking to test belief (and false belief) attribution by participants to robots. Marchesi et al. (2021) tracked the size of participants’ pupils as an indicator of surprise at unexpected results. Alternatively, Spatola et al. (2019) tested whether the presence of a humanoid robot increased performance on a Stroop task, in the way presence of other humans does. Finally, Li et al. (2022) developed a version of the Implicit Association Test designed to track implicit attributions of agency and experience to humanoid robots.

These methods show that implicit measures of anthropomorphism are possible, though they are clearly at an early stage of development. Unfortunately, the results to date do not tell a coherent story that converges on a general understanding of the processes behind implicit anthropomorphism. Additionally, although these measures get around the first limitation of explicit measures, they arguably share a second limitation.

The second limitation of explicit measures is that they generally treat anthropomorphism only as attributing a certain human-like trait to nonhuman entities. The questions in IDAQ (Waytz et al., 2010a), for example, ask whether entities possess “free will,” “intentions,” or “consciousness.” The implicit measures above operate on the same general view of anthropomorphism. Złotowski et al. (2018) test for priming based on implicit attributions of humanness to Robovie. Li et al. (2022) use their IAT variant to test for implicit attributions of agency and experience. The other measures generally test the extent to which humans treat machines as social agents. This is a valuable and informative approach, but it arguably still tests (implicit) attributions of the anthropomorphic trait of social agency. Understanding anthropomorphism as being about whether or not to attribute particular traits is common (e.g., Epley, 2018) but it misses complexities in the phenomenon. Anthropomorphism is not always about the yes/no decision of whether to attributing some trait or its absence; it can come in many forms.

Anthropomorphism can target different types of beings, such as technology, animals, spiritual agents, cartoon characters, and forces of nature. The differences matter. Measures tailored for human-machine interactions will provide less information about anthropomorphism outside that specific domain. Attributing a physical body might count as anthropomorphizing a spiritual agent (Barrett and Keil, 1996), for example, but not as anthropomorphizing a physical robot. Things get messier with nonhuman animals. Animals *do* have minds (the question is *what* sort), and many are social agents (the question, again, is what sort). Comparative (animal) psychologists have long worried about the impact of anthropomorphism on the science (e.g., de Waal, 1999; Sober, 2005; Dacey, 2017). More specifically, if we interpret the actions of animals in the same way that we would interpret those of humans, we might miss species-specific traits that are present, rather than posit traits that are not (Rivas and Burghardt, 2002; Williams et al., 2020). We might also make mistakes that have nothing to do with the presence/absence of some trait. The primate grin is a prime example. When we humans see a grin on a nonhuman primate, such as a monkey, gorilla, or chimpanzee, it looks like a big toothy smile. The animal *looks* to be happy. In reality, this “bared teeth” expression typically signals a state more like fear, anxiety, and social submissiveness (though it is likely an evolutionary homolog to the human smile; Preuschoft and van Hooff, 1997; Parr and Waller, 2006). Interpreting the animal in the way we interpret humans, in this case, leads us to get it wrong, so this is a case of interest for our understanding of anthropomorphism (e.g., Dacey, 2017). However, it is not captured by existing measures.

Here, we develop a measure of implicit anthropomorphism that avoids these two limitations, based on the primate grin example. Overall, face perception is a strong candidate for measures of implicit anthropomorphism. Faces are important social stimuli (Tsao and Livingstone, 2008). Accurately recognizing others’ emotions is essential for effective social and interpersonal functioning (Carstensen et al., 1998). As such, one might expect that processing and recognizing faces and emotions is a highly practiced skill. There is also direct evidence that, at least under some circumstances, face processing is automatic: It is fast, obligatory, and requires limited attentional resources (Palermo and Rhodes, 2010). Faces are also processed more rapidly than other visual stimuli (Bradley et al., 2000) and, in particular, faces displaying emotional expression seem to require minimal time and resources (Isaacowitz et al., 2007). Moreover, we easily see faces in everyday objects and simple patterns (even just two dots and a line:/), known as “pareidolia” (Liu et al., 2014), and apparent faces on robots make them easier to anthropomorphize (Phillips et al., 2018). Given all this evidence, it stands to reason that automatic processes responsible for face perception are major contributors to anthropomorphism.

Our method is inspired by the work of Carroll and Young (2005), who used an affective priming task. They primed participants with images of human faces expressing five of the basic emotions (happiness, sadness, fear, anger, and disgust). After priming (750 ms stimulus onset asynchrony), participants were shown a word target and asked to categorize it as belonging to one of the five emotional categories (for example: for anger, *furious*; for happy, *merry*; for sad, *miserable*; for fear, *petrified*; for disgust, *revolted*). The prime images either displayed an emotion congruent to the emotional category of the categorization task, an incongruent emotion, or a neutral facial expression as control. They found a facilitation effect such that congruent face-word pairs were responded to faster than incongruent face-word pairs. The assumption is that the face prime either facilitates or interferes with target word identification by activating processing of a related or unrelated emotion.

We modified this method to use animal, rather than human, faces. Animals (not just primates) can appear to humans to express certain emotions in multiple ways, including facial expression and body posture (e.g., Horowitz, 2009; our pilot study of stimulus images indicates the same). At least, people *report* that the animals appear to express an emotion. The question is whether they are *automatically* processing those apparent emotions in the same way they process human emotional expressions. We hypothesized that they do, and as such, we would find similar priming effects with nonhuman animal “expressions.” Schirmer et al. (2013) found that human participants recognize dog emotions better than chance, both explicitly and implicitly, using a somewhat similar priming paradigm. They primed participants with images of dogs and infants that had just experienced an event likely to cause happiness or sadness, after which participants performed a lexical decision task on words with positive or negative valence. They found congruency effects, such that words were identified faster and more accurately if they followed images congruent with the target word, compared to incongruent pairs. Because they found this effect with prime images of both dogs and human infants, the authors suggest this is evidence that human and nonhuman facial expressions are processed “similarly.” We agree this shows that they are similar in the sense that both are processed automatically, and this result lends confidence to the feasibility of our method. However, we do not take it to test anthropomorphism as directly as we intend. Schirmer et al. (2013) categorized images based on the cause of the facial expression, and as such the (presumed) *actual* emotion felt by the animal. Our test categorized images based on the *apparent* emotion as reported by participants. Therefore, Schirmer et al. tested whether humans are accurate in (implicitly) attributing animal (dog) emotions, whereas our goal is to illuminate the cues and processes they use to make those attributions. If humans attribute an emotion even when they get it wrong, it is evidence that they are interpreting the emotion based on superficial similarities in the animal’s expression to human expressions. Indeed, cases where people do get it wrong, such as the primate grin, provide the best evidence for this. If we find this in a priming task, it would provide further support for implicit anthropomorphism as a factor for misattribution of emotions to animals; interpreting the facial expressions of animals in the same way (based on the same cues) as one interprets a human face.

In addition to changing the primes from Carroll and Young (2005), we also simplified the emotion categories of the primes and targets to only positive and negative valence of “happy” and “sad.” We chose these broad categories as a starting point (as did Schirmer et al., 2013 in their experiment), due to a concern that identification of more specific categories might show variance that is too high in the early stages of development of the measure.

This measure gets around the limitations of explicit measures discussed above because, first, if the stimulus onset asynchrony is short enough, it reflects a form of automatic priming (Neely, 1977). Second, it tests a kind of anthropomorphism that has generally been ignored in the existing empirical literature; it is not about attributing or not attributing some human-likeness to the animals, because the two traits that might be attributed are plausibly equally “human-like.”

The goal is not to replace existing measures, explicit or implicit, but to supplement them. This new measure tests a different phenomenon in a different domain. It seems probable that various implicit processes contribute to anthropomorphism, some perceptual, some emotional, and some cognitive. Likely, we need a collection of implicit measures to test different aspects of anthropomorphism depending on the question being asked. Even in its implicit forms, we suggest that anthropomorphism is best understood as a complex and multifarious collection of phenomena, rather than a unitary phenomenon.

Adding further complexity, tendencies to anthropomorphize appear to vary across development. Very young children show evidence of anthropomorphism (see Geerdts, 2016, for a review) and may engage in more anthropomorphizing behaviors than adults (Epley et al., 2007). Children as young as toddlers appear to form attachments and attribute human-like behaviors and states to robots (Sharkey and Sharkey, 2011) and to toys (Gjersoe et al., 2015). At the other end of the lifespan, things are less certain. Older adults, and older women in particular, have been found to be more likely than teens and younger adults to attribute human-like qualities to robots and androids (in a sample ranging from age 15 to 70s; Kamide et al., 2013). In contrast, though, Letheren et al. (2016) found evidence for decreased anthropomorphic tendencies as age increased, with older adults being less likely to anthropomorphize than younger adults. In a meta-analysis focusing on robots and chatbots, age was negatively correlated with anthropomorphism (Blut et al., 2021). It is worth noting that these studies used questionnaire methods: Kamide et al. developed a scale specifically for examining attitudes toward robots, Letheren et al. used a brief version of the IDAQ (Waytz et al., 2010a), and Blut et al. included studies with quantitative measures in their meta-analysis. Therefore, there is conflicting evidence concerning age-related tendencies to anthropomorphize, and it has focused on explicit measures.

A fuller understanding of age-related changes will provide further evidence about how the underlying processes operate. In particular, it can provide evidence of the contributions of implicit and explicit components to anthropomorphic responses. Although aging is often associated with decreases in many cognitive processes (e.g., working memory, executive function, long-term memory; Blazer et al., 2015), automatic processes tend to be preserved. In fact, in some cases, older adults rely more than their younger counterparts on automatic processes (Jennings and Jacoby, 1993, 1997). If there are automatic components to anthropomorphism, they should be preserved in age and possibly manifest more robustly, given older adults’ increased reliance on automatic processes and their greater sensitivity to and experience with emotion processing. Therefore, a priming task might provide insight into the extent to which these automatic components change over the lifespan. Compared to younger adults, older adults also have acquired a richer and more complex knowledge base (Umanath and Marsh, 2014). This knowledge base includes interpersonal interactions; thus, one could argue that older adults have more practice at processing and recognizing emotions than younger adults (Scheibe and Carstensen, 2010). Older adults also typically attend to emotional information (in particular positively valenced information) more than to neutral information, and more than younger adults (Mather and Carstensen, 2005; see Kim and Barber, 2022, for a review). According to the socio-emotional selectivity theory (Carstensen, 2006), as a result of the aging process, older adults shift their focus toward emotion regulation and maintaining well-being. This suggests that they might be more sensitive to priming effects because of attentional direction toward emotional information. However, older adults sometimes perform worse than younger adults at emotion recognition, especially for complex verbal materials and certain facial expressions (Isaacowitz et al., 2007; Kim and Barber, 2022). This might present a complicating factor, as it suggests that older adults may be less susceptible to priming effects, if they fail to identify the emotion in the brief SOA window. Furthermore, older populations are of particular interest because of increasing interest in social robots that can assist older adults to age in place (e.g., Rashidi and Mihailidis, 2013). Though this study tests anthropomorphism of animals rather than robots, it can be a step toward a deeper understanding of how this population responds, explicitly and implicitly, to non-human agents.

Overall, comparing the development of implicit and explicit anthropomorphism through the lifespan can provide evidence about their relationship. Most obviously, if they follow opposing courses, for instance, if explicit anthropomorphism decreases while explicit anthropomorphism increases, it might suggest a process dissociation. Therefore, in addition to the priming task, we administered the IDAQ to participants to examine age differences. More generally, including an aging sample also provides generality and external validity to our results by extending the phenomenon to a different population.

Below we report the results of five experiments examining these issues. To preview our findings, although there were variations in methodologies across studies, we found consistent evidence that participants, both younger and older, are biased in their interpretations of the apparent emotional expressions of multiple types of animals and such biases lead to increased errors on word targets that were incongruent with the apparent expression.

## Experiment 1

### Participants

Target sample sizes were determined based on the sample size in Carroll and Young (2005) and on a sensitivity analysis. In their Experiment 1, 22 participants were tested with 60 trials each. Given stimulus selection constraints (see Materials) we had fewer trials; therefore, we aimed to triple their sample size at a minimum. Given the increased variability in responses in aging, we determined that approximately 90 participants in each age group would provide more stable estimates. A sensitivity analysis in G*Power 4 (Faul et al., 2007) indicated this would allow us to detect an effect size *f* of 0.08 with 0.80 power.

Ninety young adults (range 17–23; parental consent was obtained for participants under 18; 62 identified as female) from Colby College and 87 older adults (range 60–96; 62 identified as female) from the surrounding area participated. See Table 1 for demographic information. All older adults were community dwelling individuals who arranged their own transportation to the research lab. Three additional younger adults participated but their data were excluded (two required and failed to provide parental consent because they were under the age of 18 and one was accidentally tested twice). Data from one older adult were omitted because of a failure to understand the instructions. Younger participants were compensated with course credit or $5. Older adults participated as part of a longer battery of tasks and were compensated at a rate of $10/h. The protocols for all experiments reported were approved by the Institutional Review Board at Colby College.

**Table 1:** Demographic information for participants in Experiments 1–4 (standard errors in parentheses).

	Younger adults	Older adults
Experiment 1		
Age	19.30 (1.25)	70.67 (5.94)
Years of education	13.16 (1.24)	16.71 (6.65)
% Women	68.9	71.3
% Native English speakers	88.9	97.7
Experiment 2		
Age	23.02 (2.32)	63.52 (5.26)
Years of education	14.87 (4.25)	14.93 (1.96)
% Women	67.7	74.1
% Native English speakers	100	100
Experiment 3A		
Age	19.58 (1.69)	–
Years of education	13.03 (1.24)	–
% Women	67.8	–
% Native English speakers	86.1	–
Experiment 3B		
Age	19.75 (1.61)	–
Years of education	13.35 (1.44)	–
% Women	68.5	–
% Native English speakers	90.8	–
Experiment 4		
Age	19.31 (1.15)	66.35 (5.73)
Years of education	13.11 (1.13)	15.88 (2.72)
% Women	64	51.1
% Native English speakers	88	100

### Materials

Word targets were 28 items selected from the ANEW emotion word database (Bradley and Lang, 1999). Fourteen items had positive valence and 14 had negative valence. Across emotional valence, words were matched on a number of dimensions known to impact word recognition latencies (Balota et al., 2004, 2007; see Table 2). Importantly, positive and negative words significantly differed in valence, but were equally arousing and equidistant from neutral. They were further matched on length, frequency, contextual diversity, orthographic and phonological Levenshtein distance (OLD and PLD, respectively), response time and accuracy in lexical decision (from the English Lexicon Project).^1^ Finally, we matched the items using Lexical Semantic Analysis (Landauer and Dumais, 1997)^2^ from the key terms *positive* and *negative*, respectively. Thus, the words were as similar as possible in key dimensions other than valence and were similarly clustered around their respective valence.

**Table 2:** Lexical characteristics of word targets.

	Negative	Positive	*p*-value
Mean valence*	1.96	7.85	<0.001
Relative valence from mean*	3.04	2.85	0.19
Mean arousal*	5.18	5.43	0.35
Length	7.14	6.69	0.56
Frequency (HAL)^	7.80	8.53	0.27
Frequency (subtitle)^	2.58	2.87	0.38
Contextual diversity^	2.45	2.71	0.38
OLD^	2.30	2.29	0.94
PLD^	2.16	2.37	0.53
LDT RT^	657.89	618.60	0.15
LDT accuracy^	0.99	0.99	0.65
LSA (distance to negative/positive)^#^	0.04	0.05	0.61

*Values from ANEW database; ^values from English Lexicon Project; ^#^values from Latent Semantic Analysis.

The primes consisted of animal faces from multiple phyla (e.g., mammals, insects, fish). Initial stimulus selection was conducted by research assistants who conducted image searches on Google using search terms such as “happy [sad] animal faces.” An initial 169 stimuli were selected. In pilot testing, 98 participants (31 identified as female) were recruited online via Amazon Mechanical Turk rated the animals’ expressions. Participants’ mean age was 34.35 years (*SD* = 11.27, range = 20–70); 14 reported having an associate’s degree; 46 had a bachelor’s degree; 13 had a high school diploma; one had not completed high school; 16 had some college; and seven had advanced degrees. Faces were presented one at a time and participants indicated whether the animal appeared happy, sad, or was displaying no emotion. An additional “I cannot tell” option was provided. Participants rated half of the animals at their own pace and stimuli were presented in random order. The remaining faces were rated on how appealing participants found them, on a scale of 1–7 (ranging from very unappealing to very appealing). This dimension was selected to try to match the stimuli for perceived “cuteness” or liking—a factor that could potentially impact perceived emotion. We used the term “appealing” to encompass a broader range of potential reactions. Attractiveness in human faces has powerful effects on multiple judgments (Langlois et al., 2000); we aimed to control for that effect (or its analog in perception of nonhumans) here. Rating tasks were counterbalanced across participants, such that each stimulus was rated on both dimensions.

For the experimental stimuli, a subset of 21 animals that had high consistency ratings were selected. Fourteen animals that were classified as “happy” or “sad” by 90% or more of the participants were selected. As fillers, we selected seven faces classified as “no expression” (i.e., neutral). Across the three emotion categories, the stimuli were matched on perceived appeal (*p* = 0.247; means ranged from 4.63 to 5.14). Additional filler trials consisted of seven color blocks in primary colors. The fillers were included to prevent participants from becoming overly attentive to the facial expressions of the animals.

All images were resized to have approximately the same height in pixels (images were of different sizes and shapes so we avoided equating them to minimize distortions or loss of image quality) and were counterbalanced across conditions, such that across participants each prime type preceded a positive or negative word an approximately equal number of times across participants. Due to the limited number of stimuli available, participants processed three or four trials in each condition resulting from crossing target valence and prime valence. No stimuli—words or images—were repeated for a given participant. Each prime and target was only presented once to prevent changes in responses as a function of repetition. Because repetition priming effects (i.e., processing changes due to repeated exposure to a specific stimulus) are generally quite powerful (Forster and Davis, 1984; Graf and Schacter, 1985), we were concerned that repetition priming effects would potentially mask any affective priming effects, which we anticipated would be comparatively small.

To examine potential individual or group differences in anthropomorphism, participants completed the IDAQ (Waytz et al., 2010a). This measure consists of 30 questions rated on a 0 (not at all) to 10 (very much) scale. The questions assess anthropomorphic responding (e.g., *To what extent does the average fish have free will?*) or non-anthropomorphic responding (e.g., *To what extent is a river useful?*).

### Procedure

**Figure 1:**
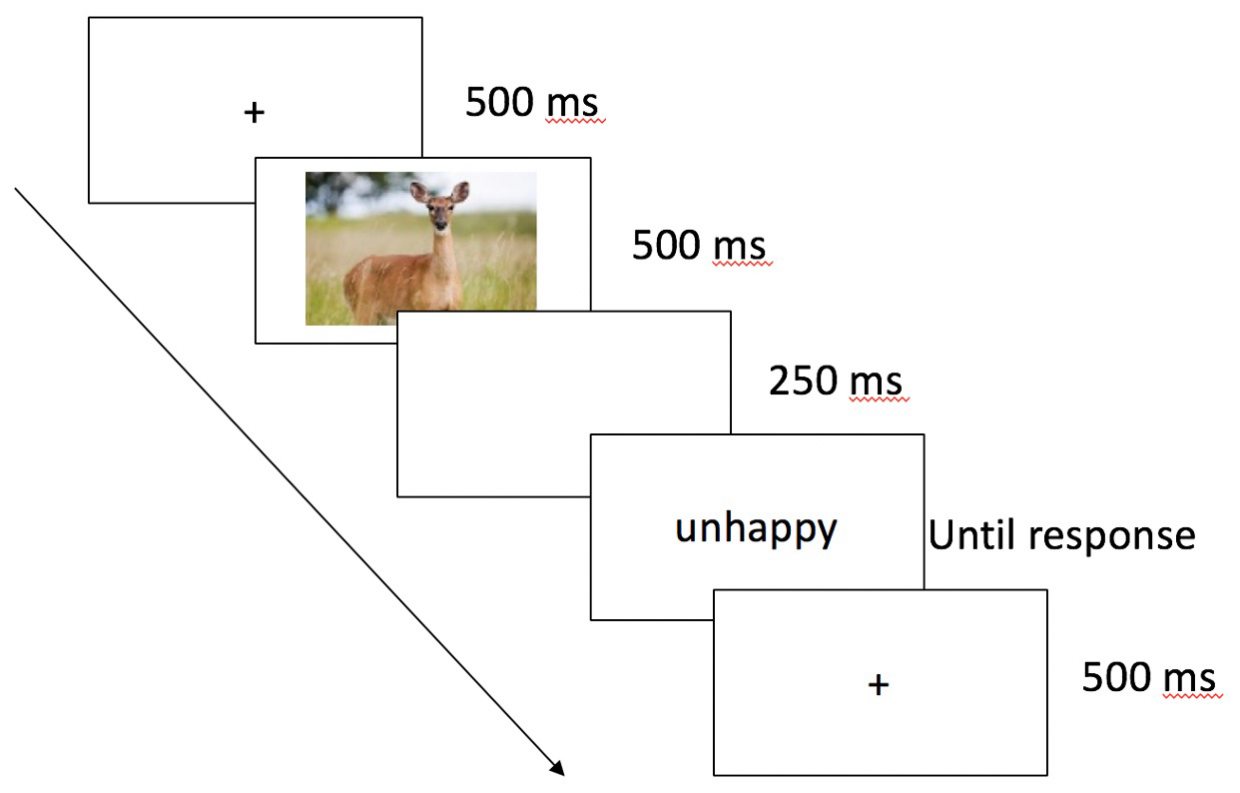
Sample trial sequence in Experiments 1, 2, and 4. Participants responded by pressing the A or K key to indicate whether the word target was a positive or negative word.

The experiment was programmed in E-Prime (Schneider et al., 2012). Participants were tested individually in a lab. After providing consent, they completed four practice trials. In both practice and experimental trials, speed and accuracy were equally emphasized. Participants were informed that they should make a judgment about the word’s valence by pressing the A or K key (positive and negative, respectively). Each trial began with a fixation cross for 500 ms. The prime—an animal face that displayed a “happy,” “sad,” or neutral expression or a color block—appeared for 500 ms and participants were explicitly told not to respond to the image. After a 250 ms interstimulus interval (resulting in a stimulus onset asynchrony, SOA, of 750 ms), the target word appeared and remained on the screen until participants made a response (see Figure 1). Image-word pairs were presented in random order. After the priming task, participants completed a surprise free recall task on the words. This task was included to provide a preliminary exploration as to whether prime-target congruency affected intentional retrieval processes. Finally, participants completed the IDAQ (Waytz et al., 2010a) and were compensated and debriefed.

## Results

In all studies, accuracy was calculated as the proportion of correct responses to the word targets (positive or negative) as a function of prime type and target type. Where relevant, a Bonferroni correction for multiple comparisons was applied and degrees of freedom corrected in cases of violations of sphericity (reported values are based on Greenhouse–Geisser corrections).

Response times were trimmed in a two-step process. First, errors were excluded and all correct responses faster than 250 ms and slower than 3,000 ms were considered outliers and removed from analyses. Next, response times that were more than three standard deviations from each participant’s mean score were removed. At the group level, we screened out participants whose mean zRT and/or accuracy exceeded the group average by more than 3 SDs (data from three younger and three older adults were omitted from analyses in this process).

In Experiment 1, step 1 of the screening process resulted in the exclusion of 296 trials (6%; errors and outliers) and step 2 resulted in an additional 119 trials (2%) being excluded. The final analyses include data from 84 older adults and 87 younger adults.

### Accuracy data

**Figure 2:**
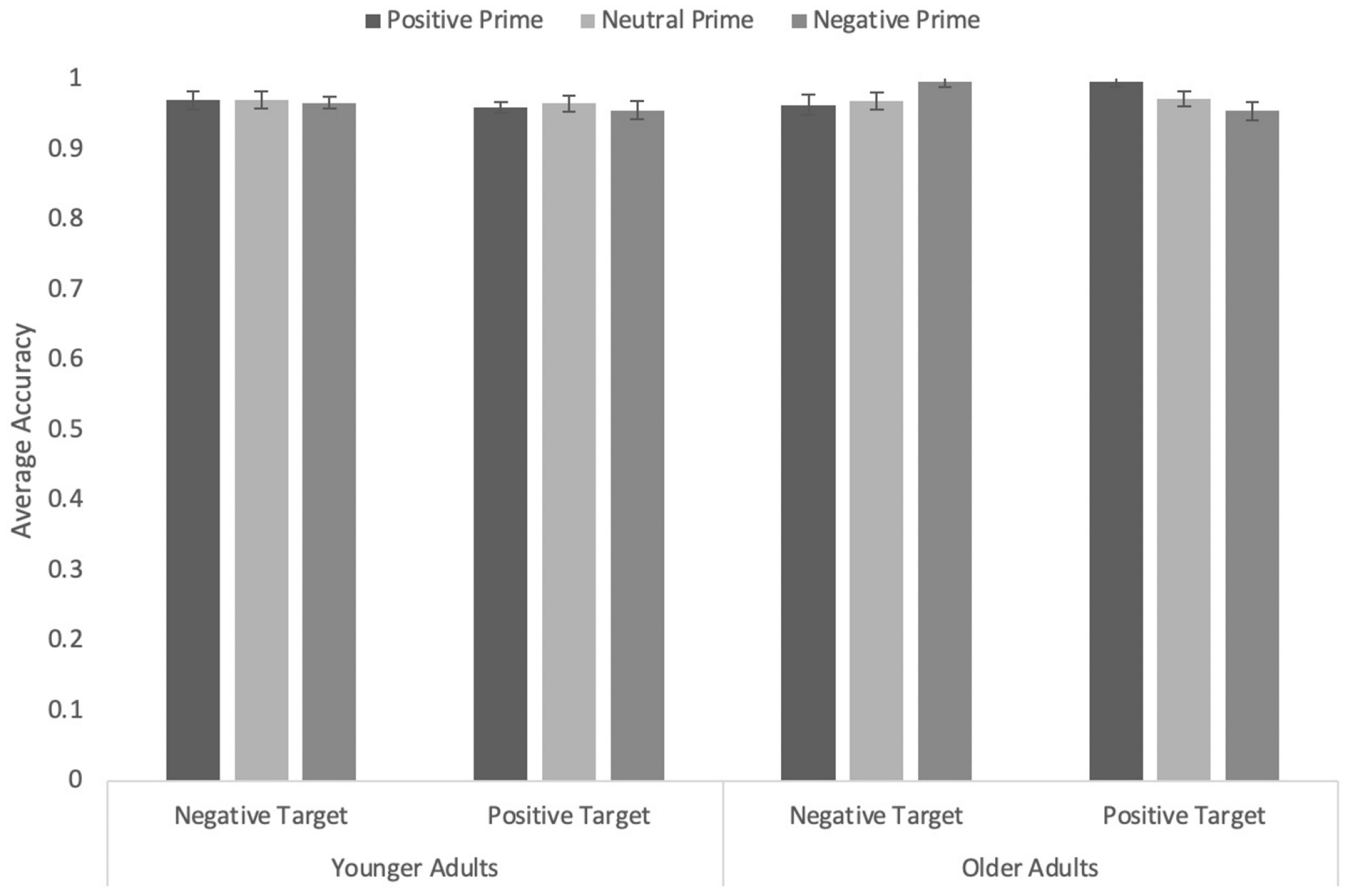
Average accuracy on the word targets in Experiment 1 with animals from multiple phyla as primes. Error bars represent standard error of the mean.

Participants’ accuracy in identifying the word valence were analyzed in a 3 (prime type: Happy vs. Neutral vs. Sad) × 2 (word valence: Positive vs. Negative) × 2 (Age: Younger vs. Older) mixed ANOVA, in which prime type and word valence were within-subjects factors and age a between-subjects factor (see Figure 2). For the sake of completeness, we first report the analyses including the neutral filler primes. However, our primary interest was in detecting any facilitation or interference due to congruity or incongruity between prime and target. The expected effects were small and the neutral trials were expected to make any effects more difficult to detect. In the analyses including all three prime types, none of the effects were reliable, all *p*s ≥ 0.084.

The ANOVA including only positive and negative primes revealed a significant interaction between prime type and word valence, *F*(1, 169) = 4.26, *p* = 0.040, η*_p_*^2^ = 0.03, reflecting a congruity effect, and a significant three-way interaction, *F*(1, 169) = 4.26, *p* = 0.040, η*_p_*^2^ = 0.03. No other effects were significant, all *F*s < 2.71, *p*s ≥ 0.102. For the sake of brevity, we focus on the three-way interaction, which we explored by examining the effect of prime type and word valence separately for younger and older adults. None of the effects were significant for younger adults, all *F*s ≤ 1.59, all *p*s ≥ 0.210. However, older adults showed a robust interaction between prime type and word valence, *F*(1, 83) = 8.4, *p* = 0.005, η*_p_*^2^ = 0.09: Responses were more accurate in congruent (happy/positive and sad/negative) than incongruent trials. Simple effects analyses indicated the effect was significant for happy primes, *F*(1, 83) = 10.19, *p* = 0.002, η*_p_*^2^ = 0.11, and marginally significant for sad primes, *F*(1, 18) = 3.87, *p* = 0.052, η*_p_*^2^ = 0.05.

In sum, happy- and sad-appearing animal faces facilitated accuracy of recognition of word valence when words were positive or negative, respectively. Importantly, however, this effect was entirely driven by the older adult participants.

### Response time data

Analyses on z-RTs including all three prime types revealed a main effect of target type, *F*(1, 165) = 18.53, *p* < 0.001, η*_p_*^2^ = 0.10, such that positive targets (*M* = −0.05, *SEM* = 0.04) yielded faster response times than negative targets (*M* = −0.16, *SEM* = 0.04; *p* < 0.001), and a main effect of age, *F*(1, 165) = 68.35, *p* < 0.001, η*_p_*^2^ = 0.29, such that younger adults (*M* = −0.40, *SEM* = 0.05) were faster than older adults (*M* = 0.19, *SEM* = 0.05). No other effects were significant, all *p*s ≥ 0.125. When only positive and negative primes were included as factors, the same pattern of results emerged.

### Free recall data

The analyses on recall data yielded a significant effect of age, *F*(1, 168) = 11.11, *p* < 0.001, η*_p_*^2^ = 0.06, such that younger adults (*M* = 0.28, *SE* = 0.01) recalled more words than older adults (*M* = 0.22, *SE* = 0.01), replicating the commonly observed episodic memory advantage for younger adults. The only other significant effect was that of target valence, *F*(1, 168) = 4.35, *p* = 0.039, η*_p_*^2^ = 0.02, such that positive targets (*M* = 0.26, *SE* = 0.01) were recalled more than negative targets (*M* = 0.24, *SE* = 0.01). None of the other effects or interactions were significant, all *F*s < 1.05, *p*s ≥ 0.352. Thus, prime-target congruency did not appear to influence more intentional, explicit retrieval processes.

### IDAQ scores

To examine whether there were any age differences in explicit measures of anthropomorphism, we examined the IDAQ scores in an age by IDAQ factor (anthropomorphism vs. control) mixed ANOVA. Overall, participants rated control items (*M* = 87.39, *SE* = 1.33) higher than anthropomorphism items (*M* = 46.53, *SE* = 1.76), *F*(1, 169) = 627.91, *p* < 0.001, η*_p_*^2^ = 0.79, and younger adults (*M* = 70.61, *SE* = 1.90) gave higher ratings than older adults (*M* = 63.30, *SE* = 1.86), *F*(1, 169) = 7.54, *p* = 0.007, η*_p_*^2^ = 0.04. The main effects were qualified by a significant interaction, *F*(1, 169) = 13.79, *p* < 0.001, η*_p_*^2^ = 0.08: Whereas ratings of control items did not differ as a function of age (*M_older_* = 86.76, *M_younger_* = 88.01), *F* < 1.0, *p* = 0.639, younger adults (*M* = 53.21, *SE* = 2.47) rated anthropomorphism items higher than older adults (*M* = 39.84, *SE* = 2.51), *F*(1,169) = 14.41, *p* < 0.001, η*_p_*^2^ = 0.08. Thus, overall, younger adults appeared to have higher explicit levels of anthropomorphism than older adults, in contrast to the sensitivity to the priming task, which revealed greater sensitivity in older adults.

We further examined whether individual differences in anthropomorphism, as captured by the IDAQ (Waytz et al., 2010a), influenced the magnitude of the priming effects. Specifically, individuals who score higher on this measure might be more likely to attribute emotions to non-human faces and therefore be more sensitive to the prime. We included IDAQ scores as a covariate and also grouped participants based on low, medium, or high scores on the anthropomorphism factor of the IDAQ and entered that variable as a factor. Neither analysis revealed any effects or interactions with the IDAQ in this or in following experiments; therefore, these analyses are not discussed further.

## Discussion

Although overall accuracy on the word valence task was almost at ceiling, the prime did affect processing: An animal face or posture that appeared to display happiness facilitated responses to words with a positive valence, whereas a face or posture that appeared to display sadness resulted in higher accuracy to negatively valanced words. Using animal faces of multiple phyla indicated that congruity effects only emerged in older adults. This is consistent with other evidence that older adults are often more attuned to emotional information than younger adults (Mather and Carstensen, 2005; Carstensen, 2006). The use of images of animals from multiple phyla, providing multiple apparent emotional indicators (face and posture) resulted in substantial variance in the prime images across a trial. In the following experiments, we limited the variety of animal faces to determine whether an effect would emerge in younger adults as well.

## Experiment 2

In Experiment 2, primes were exclusively faces of primates, which are the most similar, phylogenetically and in terms of appearance, to humans. We also made the images themselves more uniform by cropping close to the animal’s face, and excluding body posture cues. This experiment, as a result, tests more directly the “primate grin” example discussed above. As explained previously, people report that animals appear to express emotions that do not match the actual emotion that species typically expresses that way. For instance, the grin looks like happiness, but expresses fear, while other facial expressions appear like human expressions of sadness though they may not actually express it. The question here is whether the anthropomorphic processes responsible for that (mis)attribution is automatic, and, in relation to Experiment 1, whether more uniform primes depicting perhaps the strongest cases for anthropomorphism will reveal priming effects in younger as well as older adults.

### Participants

In this and subsequent studies, data collection was expanded to include an online sample. The target populations (both younger and older adults) at Colby College are too small for the purposes of the second experiment because we excluded all participants who had previously completed Experiment 1. Thus, we were unable to recruit a sufficient sample for in-person testing. Participants were tested online and were recruited from Amazon Mechanical Turk and compensated $0.80. Recruitment was limited to participants with a US IP address, an approval rating greater than 95%, and a high school education level. Ninety-six younger adults (age range 18–36; 67.7% women) and 85 older adults (age range 52–80; 74.1% women) completed the task (see Table 1). Data from an additional four participants were unusable because of missing demographic information and an additional 10 participants’ data were incomplete. All participants reported being native English speakers.

### Materials

To increase the number of trials in each cell, the color block prime trials were excluded and the number of targets increased to 30, still matched on all relevant dimensions, thus yielding five trials in each cell (prime type by word valence). Word targets were matched on all the same dimensions as those used in Experiment 1. The animal primes consisted of primate faces (e.g., chimpanzees, gorillas, orangutans, monkeys) that were selected to display the target emotional categories. As in Experiment 1, a pilot study was conducted online to ensure the selected faces represented the appropriate emotions. Thirty-three participants from Amazon Mechanical Turk rated 75 images as in Experiment 1. The participants’ average age was 33.79 (*SD* = 10.04, range 19–56); 17 identified as female and two did not report gender.

Twenty faces with high consistency of ratings (*M* = 0.84, range 0.67–0.94) were chosen to represent each category of positive and negative emotions. Positive emotions included happiness and surprise and negative emotions included sadness, fear, and anger. The rating consistency for the 10 positive faces was generally higher than for the 10 negative faces (*M* = 0.91 vs. *M* = 0.77), suggesting positive expressions were more uniformly identified and classified as such. Ten additional images were selected as neutral primes (with an average rating consistency of 0.71). Images were cropped to only show the face (i.e., all background information was removed as much as possible) and resized to be all the same size. A neutral blue background frame was placed around each image. Stimuli were counterbalanced such that each word target was preceded by a different emotional prime image across participants.

### Procedure

The experiment was programmed in Gorilla (Anwyl-Irvine et al., 2020) using the same timing parameters as Experiment 1. Participants provided consent and were debriefed online. Given the inconclusive findings in Experiment 1, the free recall task was removed and participants completed an electronic version of the IDAQ (Waytz et al., 2010a). Older adults completed the task in approximately 10 min and younger adults in approximately 9 min.

## Results

The response screening procedure resulted in the removal of 765 trials (13%) in the first step and an additional 112 trials (2%) in the second step, leaving 4,793 trials in the analyses. After removing data from participants whose average response latencies and error rates exceeded the group average for their age group, data from 80 older and 91 younger adults were available for analyses.

### Accuracy data

**Figure 3:**
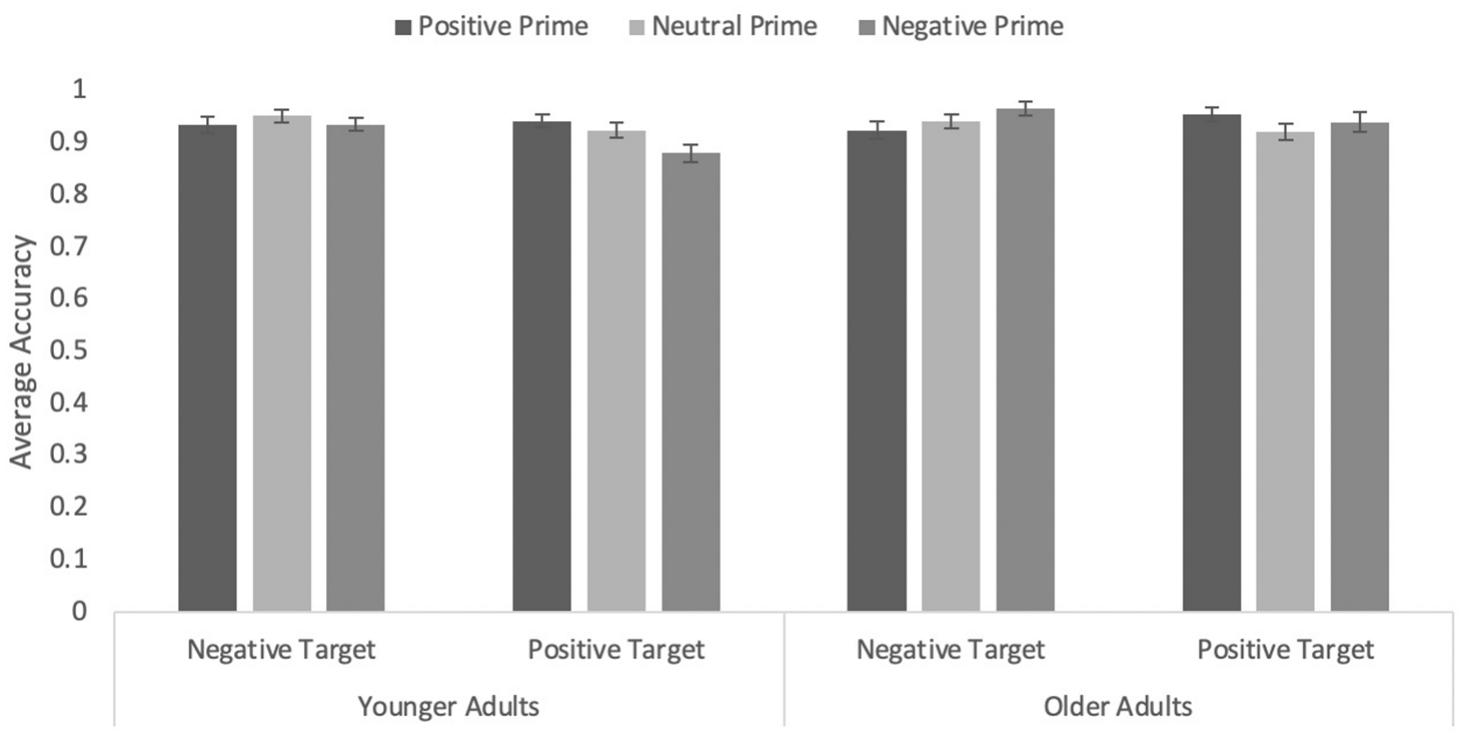
Average accuracy on the word targets in Experiment 2 with primates only as primes. Error bars represent standard error of the mean.

An ANOVA with age, target valence, and prime valence (including all three levels) on correct responses revealed an interaction between prime valence and age, *F*(1.79, 302.10) = 5.52, *p* = 0.006, η*_p_*^2^ = 0.03 (see Figure 3). Younger adults were significantly less accurate when primes were negatively valanced (*M* = 0.91, *SEM* = 0.01) than when primes were positively valanced (*M* = 0.94, SEM = 0.01, *p* = 0.009) and marginally less accurate than when primes were neutral (*M* = 0.94, SEM = 0.01, *p* = 0.069), *F*(2, 168) = 4.74, *p* = 0.010, η*_p_*^2^ = 0.05. Older adults’ accuracy did not differ as a function of prime type, *F* = 2.04, *p* = 0.13.

Critically, the prime type by word valence interaction was significant, *F*(1.843, 311.45) = 5.59, *p* = 0.004, η*_p_*^2^ = 0.03. Simple effects analyses revealed that the congruity effect was significant for negative primes, *F*(1, 169) = 10.39, *p* = 0.002, η*_p_*^2^ = 0.06, but not for positive primes, *p* = 0.226, and marginally so for neutral primes, *F*(1, 169) = 3.47, *p* = 0.064, η*_p_*^2^ = 0.02 (accuracy for negative targets was slightly higher than that for positive targets). None of the other main effects or interactions were significant, all *F*s ≤ 1.57, all *p*s ≥ 0.212. Given the effect emerged even when including the neutral primes, we do not report the focused analysis only including positive and negative primes.

### Response time data

Only one effect approached significance: Responses to positive word targets (*M* = −0.12, *SEM* = 0.03) were slightly faster than those to negative word targets (*M* = −0.07, *SEM* = 0.03), *F*(1, 166) = 3.86, *p* = 0.051, η*_p_*^2^ = 0.02. None of the other effects in response latencies were significant, all *F*s ≤ 2.42, all *p*s ≥ 0.09.

### IDAQ scores

In the ANOVA examining IDAQ scores as a function of factor (anthropomorphic vs. non-anthropomorphic) and age, only the effect of factor was significant, *F*(1, 165) = 610.88, *p* < 0.001, η*_p_*^2^ = 0.79; participants rated control items (*M* = 91.27, *SE* = 0.132) higher than anthropomorphism items (*M* = 46.89, *SE* = 0.191). Neither the effect of age nor the interaction was significant, *F*s < 1.0, *p*s ≥ 0.697.

## Discussion

Once again, the analyses revealed that processing of emotionally valanced words can be affected by a non-human face displaying an apparent emotional expression. One key difference from Experiment 1 is that the effect also emerged in younger adults and not only in older adults. This suggests that when the animal faces are more similar to human faces and primes are more uniform, participants in both age groups were affected by the perceived emotional expression. The fact that neutral primes also yielded a difference in accuracy for positive vs. negative targets is possibly due to the relatively low consistency rate for emotion identification for these primes. Thus, to some participants, the neutral faces might have appeared more negative. Although speculative—in particular for primates—a neutral expression might convey anger or displeasure.

## Experiment 3

In the next two experiments, we explored the time course of the congruity effects. Specifically, given that the effect in general seemed to be more robust in older adults, who showed robust congruity effects for both primate faces and multi-phyla faces, we speculated that additional processing time might allow the effect to emerge in younger adults more robustly. To slow down response times, we implemented a visual degradation manipulation of the word target to increase reading difficulty in Experiment 3A. As a direct contrast to a slowing procedure, in Experiment 3B, we imposed a speeded response deadline to force a more rapid response. Thus, across two experiments, we attempted to isolate speed of responses. If additional processing time allows emotion recognition to emerge more fully, the slower response latencies might increase the magnitude of the effect. In contrast, speeding responses might eliminate the effect. Because the manipulations significantly increased the difficulty of the task, these two experiments only included younger adult participants.

### Experiment 3A

#### Participants

Given the increased task difficulty, we oversampled relative to the previous experiments to account for lost data. One hundred and twenty-eight younger adult participants (age range 17–24; 87 identified as female) completed the study. Data from seven participants were unusable due to a programming error. Of the remaining 121 participants, 82 were Colby College students (27 tested in the lab, 54 tested remotely) and 40 were recruited from the online platform, Prolific (prolific.co). None of the Colby College students had participated in Experiment 1. Colby students received course credit and participants on Prolific were paid $1.07 (average $8.35/h). See Table 1 for demographic information.

#### Materials

The same stimuli used in Experiment 2 were used in this study.

#### Procedure

Consent and debriefing were administered online. Prime presentation was the same as in Experiment 2. The target was degraded using a flicker paradigm; a string of characters (e.g., #$%) the same length as the target alternated with the word target at a rate of 34 ms until participants made a response. Participants completed six practice trials before the main experimental trials. In all other aspects, the procedure was the same as Experiment 2.

## Results

The screening process resulted in the removal of 680 trials (18%) in step 1 and 23 additional trials in step 2 (less than 1%). Given the increased task difficulty, it is not surprising that more trials resulted in errors or in excessively long response latencies. Data from six participants were omitted from analyses because their error rate or average response latency exceeded the group mean by more than three standard deviations, leaving 115 complete data sets.

### Accuracy data

The ANOVA with prime type and word valence revealed a main effect of word valence, *F*(1, 228) = 14.33, *p* < 0.001, η*_p_*^2^ = 0.11, reflecting higher accuracy for positively valenced targets (*M* = 0.92, *SEM* = 0.013) than for negatively valenced targets (*M* = 0.87, *SEM* = 0.01).

**Figure 4:**
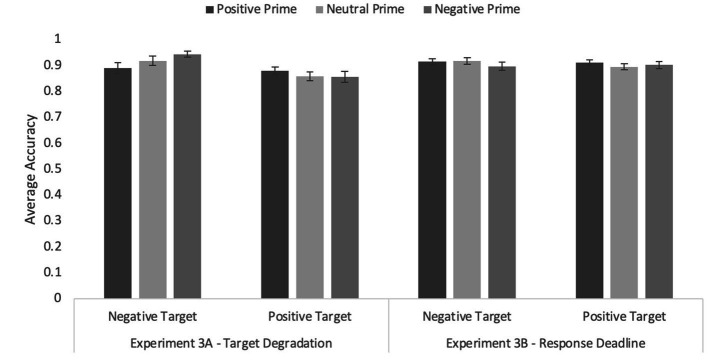
Average accuracy on the word targets in Experiments 3A (target degradation) and Experiment 3B (response deadline) with primates only as primes. Error bars represent standard error of the mean.

Once again, there was a significant prime type by word valence interaction on accuracy, *F*(1.79, 204.62) = 3.78, *p* = 0.041, η*_p_*^2^ = 0.03 (see Figure 4). Tests of simple effects revealed a significant congruity effect when primes were negatively valenced, *F*(1,114) = 16.49, *p* < 0.001, η*_p_*^2^ = 0.13, but not when primes were positively valenced, *F* < 1.0, *p* = 0.667. Interestingly, neutral primes also resulted in higher accuracy for negative than positive targets, *F*(1, 114) = 7.85*, p* = 0.006, η*_p_*^2^ = 0.06. The effect of prime type was not significant, *F* < 1.0, *p* = 0.433.

### Response time data

None of the effects were reliable in response times, all *F*s ≤ 1.03, *p*s ≥ 0.313.

To address potential differences in test environment, we re-analyzed the data, accounting for the test environment. There were three distinct groups of participants: Colby students tested in the lab (*n* = 27), Colby students tested remotely (most of whom were on campus; *n* = 53), and participants from Prolific (*n* = 35). There were no differences among groups in overall accuracy, *F*(2, 112) < 1.0, *p* = 0.518, or in z-transformed response times, *F*(2, 112) < 1.0, *p* = 0.627. When participant group was included in the ANOVA examining accuracy as a function of prime valence and of target valence, the three-way interaction was not significant, *F*(4, 224) < 1.0, *p* = 0.479, suggesting the congruity effects were similar across samples. We acknowledge, however, that the small number of participants tested in person, relative to those tested online, reduces the power to detect small effects.

## Discussion

The results of Experiment 3A largely mirrored those of Experiment 2: Congruity effects in younger adults appear to be driven by the negatively valenced primes. Neutral primes also affected target processing, in this experiment and marginally in Experiment 2. It is possible that neutral images were perceived as slightly negative, especially in contrast to the positive expressions, a point we return to in the General Discussion. The effect size observed in Experiment 2 was smaller than that observed in Experiment 3A (η*_p_*^2^ = 0.08 vs. η*_p_*^2^ = 0.13, respectively), suggesting the degradation manipulation did indeed boost the effect.

### Experiment 3B

#### Participants

As in Experiment 3A, we oversampled to account for the expected increase in lost data. One hundred and thirty-three young adults (age range 17–24; 91 identified as female) completed the task; an additional nine started but did not complete it. Of the completed data sets, 93 were students at Colby College (26 tested in person, 67 tested online) and 40 were recruited on Prolific (see Table 1). Students received course credit for their participation; participants on Prolific were paid $1.07 (average $10/h).

#### Materials

The same stimuli were used in Experiment 3B as in Experiment 3A.

#### Procedure

The same general procedure used in Experiment 2 was implemented. The key difference was that participants were told to respond quickly. Instructions specified they would have less than 1 s; after 800 ms, if no response was detected, a message informing participants that they were too slow and to try to respond faster appeared on the screen until a response was detected. Targets were presented as in Experiment 2 (i.e., there was no degradation).

## Results

The screening process resulted in the removal of 440 trials (11%) in step 1 and 47 additional trials in step 2 (1%). Data from an additional three participants were omitted from analyses because their error rate or average response latency exceeded the group mean by more than three standard deviations, leaving 130 complete data sets.

### Accuracy data

In the accuracy data, no effects were significant, all *F*s ≤ 1.0, *p*s ≥ 0.410 (see Figure 3). Overall accuracy was 0.91 (*SEM* = 0.01), suggesting that even under speeded conditions, participants were able to perform the task.

### Response time data

The response latency data only revealed a significant effect of target valence, *F*(1, 129) = 13.90, *p* < 0.001, η*_p_*^2^ = 0.10, such that positively valenced words (*M* = −0.12, *SEM* = 0.02) were responded to more quickly than negatively valenced words (*M* = 0.009, *SEM* = 0.02). Neither the effect of prime type nor the interaction was significant, both *F*s ≤ 1.0, *p*s ≥ 0.770.

To verify that the deadline manipulation was indeed effective in speeding participants’ responses and that the degradation resulted in slower RTs, we calculated the average response times across all valid trials in Experiments 2, 3A, and 3B. In Experiment 2, overall average latencies were 781 ms (*SD* = 190), in Experiment 3A, RT averages were 1,063 ms (*SD* = 325), and in Experiment 3B, they were 618 ms (*SD* = 68). These differences were significant, *F*(2, 336) = 128.32, *p* < 0.001, η*_p_*^2^ = 0.44; all pairwise comparisons were significant (all *p*s ≤ 0.001). Thus, it appears the differential manipulations were indeed effective at achieving the goal of selectively speeding up and slowing down responses.

As in Experiment 3A, we examined whether there were any systematic differences between population groups. None of the effects including participant group as a factor were significant: In accuracy, all *F*s ≤ 1.48, *p*s ≥ 0.230; in response times, all *F*s ≤ 1.80, *p*s ≥ 0.129.

## Discussion

To summarize, across all three experiments, we found evidence for small but replicable emotion priming effects from animal faces. Older adults consistently showed congruity effects: Higher accuracy in categorizing a word’s valence as positive or negative when the word was preceded by an animal displaying a happy or sad expression, respectively, compared to when the prime and target represented conflicting emotional expressions. Younger adults seemed more selective in their sensitivity to the effect: They only showed priming effects when primate faces were used and exclusively for negative targets. Based on the results of Experiment 3B, the effect appears to emerge later in the processing of the target words; speeding responses eliminated any congruity effects.

## Experiment 4

In experiment 4, we replaced the animal prime stimuli with human faces. This serves as a control to the tests above, demonstrating whether the effects of emotion perception in human faces are directly comparable to emotion perception in animal faces. This also serves as a manipulation check to verify that our novel results—affective priming from animal faces to word targets—emerges under more standard conditions (*cf.* Carroll and Young, 2005). Due to the COVID-19 pandemic, data collection was conducted entirely online. Therefore, successfully replicating the patterns from Carroll and Young and the experiments reported here in an online format gives more confidence in the data.

### Participants

Younger adults (*n* = 100, age range 17–22; 64 identified as female) were recruited from Colby College’s psychology participation pool and completed the study online. Ninety-four older adults (age range 60–92; 48 identified as female) were recruited on Prolific (see Table 1 for demographic information). All participants completed the task at a time and place of their choice.

### Materials

Faces were selected from the Chicago Face Database (Ma et al., 2015). A total of 30 faces were selected; these included Black and white men and women. The faces were of mostly younger adults because of increased difficulties in emotion recognition in older faces (Fulton and Bartlett, 1991). An equal number of faces displayed positive emotions (i.e., happiness), neutral emotions, and negative emotions (e.g., sadness, anger) to mimic the animal stimuli used in prior experiments. The same word stimuli from Experiment 2 were used.

### Procedure

The procedure was the same as in Experiment 2; the study was conducted entirely online. Given the brevity of the task (most participants finished in under 10 min), we had limited concerns about attrition or task interruptions. Consent and debriefing were administered online.

## Results

The data were processed as in the previous studies. Initially, 454 trials (errors and extreme RTs; 16%) were removed. An additional 58 trials (2%) were removed in the second stage. Data from three older and six younger participants were omitted from the analyses due to deviation scores (in accuracy and response latency from the group average that exceeded 3 standard deviations).

### Accuracy data

Correct responses were submitted to the same prime valence by target valence by age ANOVA as in Experiment 2. Overall, older adults (*M* = 0.94, *SEM* = 0.01) were more accurate than younger adults (*M* = 0.91, *SEM* = 0.01), *F*(1, 183) = 3.93, *p* = 0.049, η*_p_*^2^ = 0.02. There was a marginal main effect of prime type, *F*(1.89, 346.49) = 3.05, *p* = 0.051, η*_p_*^2^ = 0.003, although none of the pairwise comparisons were significant (all *p*s ≥ 0.091). Target valence interacted with age, *F*(1, 183) = 4.35, *p* = 0.038, η*_p_*^2^ = 0.02. Whereas older adults’ accuracy did not differ as a function of target valence, *F*(1, 183) < 1, *p* = 0.415, younger adults were more accurate when targets were negative (*M* = 0.92, *SEM* = 0.01) than when targets were positive (*M* = 0.89, *SEM* = 0.02), *F*(1, 183) = 4.60, *p* = 0.033, η*_p_*^2^ = 0.03.

**Figure 5:**
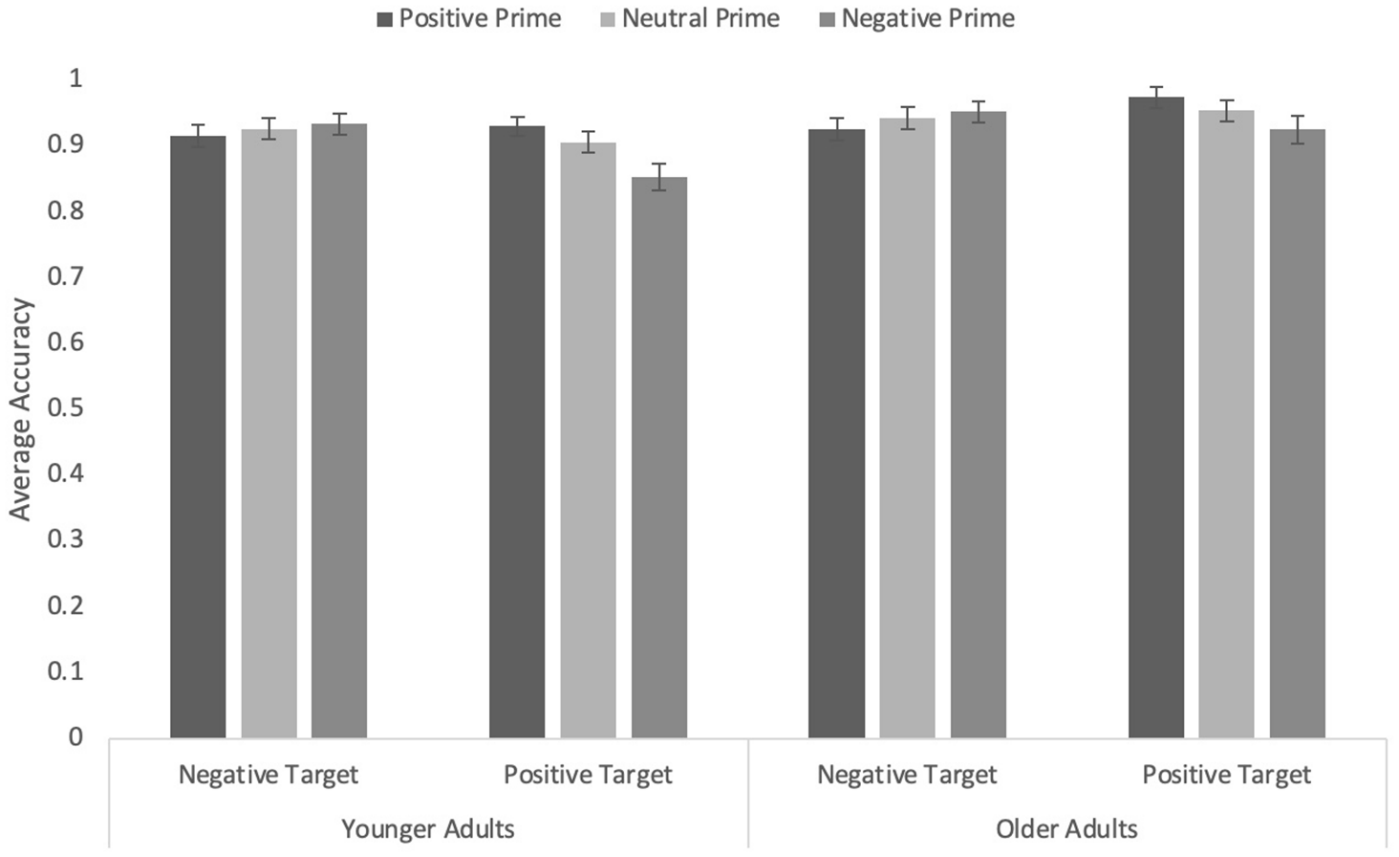
Average accuracy on the word targets in Experiment 4 with human faces as primes. Error bars represent standard error of the mean.

Critically, as in previous experiments, the prime valence by target valence was significant, *F*(1.86, 340.27) = 14.01, *p* < 0.001, η*_p_*^2^ = 0.07 (Figure 5). When primes were positively valenced, responses to positive targets were more accurate than those to negative targets, *F*(1, 183) = 5.99, *p* = 0.015, η*_p_*^2^ = 0.03. When primes were negatively valenced, responses to negative targets were more accurate than those to positive targets, *F*(1, 183) = 15.29, *p* < 0.001, η*_p_*^2^ = 0.08. Thus, a robust congruency effect was observed when the primes were human faces. When primes were neutral, there was no difference in accuracy as a function of target valence, *F* < 1.0, *p* = 0.757. No other effects or interactions were significant, all *F*s ≤ 1.0, *p*s ≥ 0.358.

### Response time data

Analyses on standardized RTs only revealed a main effect of target valence, *F*(1, 177) = 5.03, *p* = 0.026, η*_p_*^2^ = 0.03, reflecting faster responses to positive (*M* = −0.12, *SEM* = 0.01) than to negative (*M* = −0.07, *SEM* = 0.01) words. No other effects were significant, all *F*s ≤ 1.91, *p*s ≥ 0.149.

In sum, the results of Experiment 4 with human faces as primes extend and confirm the findings of the previous studies: Congruity effects emerged in accuracy and not in response times. When a briefly presented face—primate or human—preceded a word target, participants categorized the word as positive or negative more accurately when the emotional expression matched the word’s valence. In contrast to Experiments 2 and 3A, neutral primes did not affect target processing. This suggests that interpreting emotions—or the lack thereof—might be a less ambiguous task when the faces are of humans.

### IDAQ scores

The age by factor analyses on IDAQ scores revealed a similar pattern to that observed in Experiment 1. Younger adults (*M* = 75.01, *SE* = 1.74) gave higher ratings than older adults (*M* = 62.90, *SE* = 1.78), *F*(1, 178) = 27.67, *p* < 0.001, η*_p_*^2^ = 0.14. Control items (*M* = 89.87, *SE* = 1.14) received higher ratings than anthropomorphism items (*M* = 47.04, *SE* = 1.73), *F*(1, 178) = 773.17, *p* < 0.001, η*_p_*^2^ = 0.81. The interaction between age and IDAQ factor was significant, *F*(1, 178) = 20.27, *p* < 0.001, η*_p_*^2^ = 0.10. For control items, younger adults (*M* = 92.96, *SE* = 1.59) gave higher ratings than older adults (*M* = 86.78, *SE* = 1.63), *F*(1, 178) = 7.33, *p* = 0.007, η*_p_*^2^ = 0.04. Younger adults (*M* = 57.06, *SE* = 2.42) also rated anthropomorphism items higher than older adults (*M* = 37.02, *SE* = 2.47), *F*(1, 178) = 33.57, *p* < 0.001, η*_p_*^2^ = 0.16. The interaction reflected the fact that the difference in ratings was larger for anthropomorphism than for control items.

### Combined analyses

Given the similarity in methodologies across experiments, we conducted some additional analyses combining the data from Experiments 1 and 2 to examine affective priming from non-human faces more closely (we omitted Experiments 3A and 3B because only younger adults were tested and due to the timing parameter changes). In Table 3, we provide a summary of the analyses across all four experiments. The substantially larger sample size provides a more stable estimate of the effects. In particular, we were interested in whether the larger, combined sample might increase the stability of the response time data.

**Table 3:** Summary of main effects and interactions in Experiments 1–4.

	Experiment 1	Experiment 2	Experiment 3a	Experiment 3B	Experiment 4	Combined analyses of Experiments 1 and 2
Main effect of target valence	No	*Marginal:* negative more accurate than positive	*Yes*: positive > negative	No	No	*Yes*; negative more accurate than positive
Main effect of prime valence	No	No	No	No	Marginal (no significant pairwise comparisons)	No
Main effect of age	No	No	N/A	N/A	*Yes*; OA more accurate than YA	No
Target by age interaction	No	No	No	No	*Yes*; YA more accurate for negative than positive targets; no difference for OA	No
Prime by age interaction	No	*Yes*; for OA, no effect of prime valence, for YA negative primes less accurate than positive or neutral	N/A	N/A	No	*Yes*; for OA, no effect of prime valence, for YA negative primes less accurate than positive or neutral
Prime by target interaction	Yes^*^: Congruent > Incongruent	*Yes*: Significant congruity effect for negative primes; marginal difference in accuracy for neutral primes (negative targets > positive targets)	*Yes*: Significant effect of congruity for negative primes; no effect for positive primes; significant difference in accuracy for neutral primes (negative targets > positive targets)	No	*Yes*; congruity effect for both positive and negative primes, no difference for neutral primes	*Yes*: Significant congruity effect for negative primes
Age by prime valence by target valence interaction	Yes^*^: Congruity effect only in OA; significant effect for positive primes; marginal effect for negative primes	No	N/A	N/A	No	No

^*^Only when excluding neutral prime trials.

Analyses on the combined data from Experiments 1 and 2 largely were consistent with the analyses on the individual data sets. An overall main effect of target valence emerged, *F*(1, 340) = 4.37, *p* = 0.037, η*_p_*^2^ = 0.01, such that negative targets (*M* = 0.96, *SEM* = 0.005) were identified more accurately than positive targets (*M* = 0.95, *SEM* = 0.005). The prime valence by age interaction observed in Experiment 2 was also seen in the combined data, *F*(2, 680) = 4.68, *p* = 0.010, η*_p_*^2^ = 0.01: For older adults, prime valence did not affect overall accuracy (*p* = 0.179), whereas younger adults were more accurate when primes were negative than when they were positive (*p* = 0.078) or neutral (*p* = 0.026), *F*(2, 339) = 0.389, *p* = 0.021, η*_p_*^2^ = 0.02. The interaction between prime valence and target valence was also reliable, *F*(1.77, 600.84) = 8.51, *p* < 0.001, η*_p_*^2^ = 0.02: A robust congruity effect emerged, but only for negative primes, *F*(1, 340) = 16.66, *p* < 0.001, η*_p_*^2^ = 0.05. None of the other effects were reliable, all *F*s ≤ 2.42, *p*s ≥ 0.121.

However, even with the larger sample, only two effects emerged as reliable in the analyses on response latencies: Younger adults (*M* = −0.24, *SEM* = 0.03) were faster than older adults (*M* = 0.05, *SEM* = 0.03), *F*(1, 333) = 53.05, *p* < 0.001, η*_p_*^2^ = 0.14, and positive targets (*M* = −0.14, *SEM* = 0.02) were identified more rapidly than negative targets (*M* = −0.06, *SEM* = 0.02), *F*(1, 333) = 18.58, *p* < 0.001, η*_p_*^2^ = 0.05. Therefore, the congruity effect reported appears to be isolated to accuracy, at least under present conditions.

## General discussion

These results indicate, first and foremost, that there is an automatic component to anthropomorphism, at least in the recognition of apparent facial expressions of non-human animals (Złotowski et al., 2018; Li et al., 2022). Participants showed similar affective priming for ape facial expressions as they did for human facial expressions. Older adults also showed priming for apparent emotional expression by a broader range of species with less uniform images. With nonhuman primes, the effects were only apparent in accuracy, not in response time (*cf.* Carroll and Young, 2005). Our tests of degraded target stimuli vs. response deadlines indicate that the congruity effect emerges more robustly and consistently with additional time to process the target stimuli in younger adults. The stimulus onset asynchrony of 750 ms is still in the range generally attributed to automatic priming, though it is at the upper end (Neely, 1977); however, when we forced participants to respond more quickly, the priming effects were eliminated. Overall, this suggests contributions from processes outside of intentional control. Future work could examine the boundary conditions of such automatic processes in terms of speed, obligatory nature, and dependence on attention (*cf.* Palermo and Rhodes, 2010).

In Experiment 1, we only found an effect of prime-target congruency in older adults. This makes sense, given that older adults are known to attend more to emotional information than younger adults (Mather and Carstensen, 2005). It is still worth pulling apart two possible reasons that the effect in this experiment was diminished relative to Experiment 2. Experiment 1 included many different kinds of animals that were rated by pilot participants as displaying positive or negative emotions for many reasons, including expression and posture (we did not ask for explanations, but pilot participant responses matched researcher intuitions closely here). As such, the animals were oriented to the camera differently, were different sizes in the image, and backgrounds were nonuniform. One possibility is simply that this lack of uniformity introduced noise that washed out any effect in younger adults. If this is the right explanation, the main priming effect found with primate primes might generalize to other species, if we could generate more uniform images of them. A second possibility is that this form of implicit anthropomorphism diminishes as one moves away from humans on the phylogenetic tree, and animals look less and less like us. This would make sense, as being “like us” seems to be an important factor for anthropomorphism in other contexts (Eddy et al., 1993; Morewedge et al., 2007). However, we will note that participants in the study piloting materials did not show this effect: when asked to give explicit ratings of whether animals appeared to show an emotion, participants freely attributed them across taxonomy. In fact, if anything, familiarity seemed more important, as familiar pets like cats and dogs were rated as more expressive than primates (see also Morris et al., 2012). So, if implicit anthropomorphism diminishes more rapidly than explicit anthropomorphism when applied to more various and more distant species, it suggests a possible dissociation between the two. Further work will be necessary to explore this difference.

As noted, older adults, relative to younger adults, appeared to be more susceptible to the primes, at least when the primes included a diverse array of species. However, other than in Experiment 1, the congruity effects were similar across age groups. Because of age-related cognitive slowing (Salthouse, 1996), it was possible that older participants would not be able to fully process the image primes due to the relatively short stimulus onset asynchrony and thereby show reduced priming effects. However, if older adults can capitalize on prior experience and knowledge (Umanath and Marsh, 2014) and on their biases to attend to emotional information (Kim and Barber, 2022), this might facilitate their processing of emotional content and allow them to compensate for the slower processing. Another possibility was that, because of impoverished emotion recognition abilities (Isaacowitz et al., 2007; Ruffman et al., 2008), older adults might show less priming—or reduced congruency effects—due to errors in emotion identification of the primes. Again, such an effect did not seem to occur. The fact that participants were asked to categorize the word targets as positive or negative, rather than to perform a more fine-grained analysis of specific emotion categories (e.g., anger, sadness, fear), likely made the task somewhat easier.

We noted above that it would be valuable to compare implicit and explicit measures of anthropomorphism with aging. Our results suggest that older adults may be more susceptible to these priming effects, but only in one of the experiments. Further, the results to date on aging with explicit measures are equivocal (Kamide et al., 2013; Letheren et al., 2016; Blut et al., 2021). In our own work, in two of the three experiments that included both age groups, older adults had lower IDAQ anthropomorphism scores than younger adults (in the other experiment, there was no difference). If the pattern holds that older adults show increased anthropomorphism on implicit measures, but decreased anthropomorphism on explicit measures, it might provide evidence of different underlying processes (e.g., Złotowski et al., 2018) which follow different courses in aging. However, neither the age difference in priming effects nor the age differences in IDAQ scores were consistent across our experiments. Given this unreliability, along with the equivocal existing literature, it is too early to draw any conclusions about the age courses of either measure.

In addition to comparing IDAQ scores across age groups, we also tested whether individual differences in IDAQ scores would predict individual differences in the magnitude of priming effects. We found no correlation, positive or negative. It appears that explicit measures of anthropomorphism, such as IDAQ, do not modulate our implicit measure of anthropomorphism. While interpreting null results is extremely difficult, if explicit and implicit measures are independent (that is, if this lack of correlation holds up to further testing) dual-process views of anthropomorphism (Złotowski et al., 2018) are better positioned to explain that result than would be any view that takes the two types of measures to probe a single psychological construct. In both age-group comparisons and individual difference comparisons, our results may suggest distinct processes underlying implicit and explicit anthropomorphism, though the results are far from conclusive.

Although we generally observed priming congruency effects, the effect was more consistent with negative primes compared to positive primes. There is evidence that negative stimuli, especially threatening ones, draw resources and attention, which, from an evolutionary standpoint, is an adaptive response (Vuilleumier, 2002). The processing of threatening stimuli is preserved in older adults (Mather and Knight, 2006). Although older adults tend to show a bias toward remembering positive information, this does not preclude processing of negative stimuli. Thus, it is possible that the negative primes were processed more rapidly due to relative shifts in attention capture. As noted in Experiments 2 and 3a, neutral primes also affected target processing: Negative targets were identified faster following neutral primes compared to positive targets. A plausible explanation is that some of the faces that were selected to be neutral were perceived as negative by participants. Interestingly, this effect only occurred when the primes were primates, not when they included animals of multiple species or humans. Perhaps neutral expressions in some primates appear to be negative or threatening, and therefore elicit facilitation for negative word targets.

Another important question is the extent to which these priming effects represent the *attribution of* a certain emotion to the animal in the image as opposed to the participants simply *feeling* that emotion themselves. While this may be a difficult distinction to prize apart in rapid automatic processing, we think there is good evidence of attribution here. The prime images, especially in Experiment 1, could have been perceived as very cute, especially the smiling ones (for example, we had a number of puppies/dogs and one very endearing manta ray). Viewing images like this may make people feel good, which might influence the categorization of targets. In our piloting of those materials, we asked participants to rate them in the apparent emotion felt, but also on how appealing participants found the image to be. The responses on this “appealingness control” were not valenced in the same way as the attributed emotions; most saliently, some of the images of sad-looking animals were also rated as very appealing, and participants reported them as making them feel very good. We did match stimuli across emotional categories on this factor, to rule out a confound between emotion identification and affective response. It is worth noting that, although the sample of images was quite small, the highest rated animals we used as primes were mostly “domestic” pets (e.g., cats, dogs, rabbits). Primates – at least in the sample reported here – were rated somewhat lower. Therefore, primates appear to be less appealing than other types of animals.

Moreover, the fact that these results match, in broad strokes, the Carroll and Young (2005) results is reason to think that the recognition of emotions here is categorical. Carroll and Young found their priming effects for five emotional categories, not just positive/negative. We simplified the categories to that single dimension because animal expressions would not likely match human expressions at such a fine grain. This simpler, one-dimensional measure may itself be more susceptible to interference based on the participants’ mood. However, if we consider Experiment 4, Experiment 2, and Experiment 1 as a sequence that increasingly moves away from the original Carroll and Young experiment, we see diminishing effect sizes, but no evidence of a categorical change. This, together with the pilot data, suggests to us that the most plausible interpretation is that these priming effects indicate a genuine implicit attribution of emotion to the animals in the images. An additional factor, that we did not test here, was the difference in response modality between key press, as was done here, and verbal responses, as was done in Carroll and Young’s work.

### Limitations and future directions

Although the congruity effect we obtained was small, it was replicated across participants and stimuli. One open question is why the effect only emerged in accuracy and not in response times. Typically, response latencies are sensitive to priming effects and to changes in the accessibility of related information following a prime. One possibility is that, because of stimulus constraints, we had a small number of observations, thereby limiting our power to detect an effect in response times, which can be somewhat variable. Even when we combined the data from Experiments 1 and 2, no effect on response times emerged. Future studies could potentially use different classes of stimuli, such as computer-generated animal faces or emojis, to increase the number of trials. Another possibility is that the effect did not emerge in response times because of the relative simplicity of the task: A dichotomous positive/negative decision might have been relatively easy. With a larger stimulus set, a more fine-grained analysis of emotion categorization and priming effects could be examined. Similarly, it would be helpful to test more uniform images of animals across taxa than those used in Experiment 1. This could help to test whether the lack of effect for young adults in that experiment is the result of noise from the stimuli, or reflects a real difference in implicit anthropomorphism of different species.

We noted in the Introduction the need for implicit measures of anthropomorphism. The results here indicate that this affective priming paradigm has some promise in that role. There are many ways this could potentially be extended. More systematic comparisons of explicit and implicit anthropomorphism would help to characterize their similarities, differences, and interrelations. This could include more testing of whether or not they follow similar trajectories with participant age, and with variation in stimulus materials (e.g., different animal species, robots, etc.). It could also be tested whether this priming effect can be manipulated independently of explicit measures, as Złotowski et al. (2018) unsuccessfully attempted with their priming effect. There are a number of factors known to influence explicit anthropomorphism that could be used here. Most obviously, these include various manipulations used to test Epley et al. (2007) three-factor model (e.g., Epley et al., 2008; Waytz et al., 2010c). On another track, given that this experiment is inspired by work in comparative psychology, it might provide a useful test of that field’s preferred control of anthropomorphism: a methodological principle known as Morgan’s Canon, which dictates that researchers prefer the hypothesis positing the simpler process (Morgan, 1894; see also de Waal, 1999; Sober, 2005). It is hard to see how this would impact implicit anthropomorphism in cases such as emotion recognition (e.g., Dacey, 2017), but given the centrality of Morgan’s Canon, it is worth gathering data about the impact it may (or may not) have on implicit forms of anthropomorphism.

The insight that implicit anthropomorphism operates in many ways in different stages of processing implies that there can be no single measure that captures it fully. However, this paradigm shows some promise in capturing important parts of it, and as such, warrants further testing.

## Data availability statement

The raw data supporting the conclusions of this article will be made available by the authors, without undue reservation.

## Ethics statement

The studies involving human participants were reviewed and approved by the Colby College Institutional Review Board. The patients/participants provided their written informed consent to participate in this study.

## Author contributions

MD: conceptualization, methodology, writing, reviewing, and editing. JC: conceptualization, methodology, writing, analyses, editing, funding. All authors contributed to the article and approved the submitted version.

## Funding

This work was supported by funding from a James McDonnell Foundation *Understanding Human Cognition* Grant awarded to JC (#220020426). The funding agency had no input on the study design, data analysis, or writing.

## Conflict of interest

The authors declare that the research was conducted in the absence of any commercial or financial relationships that could be construed as a potential conflict of interest.

## Publisher’s note

All claims expressed in this article are solely those of the authors and do not necessarily represent those of their affiliated organizations, or those of the publisher, the editors and the reviewers. Any product that may be evaluated in this article, or claim that may be made by its manufacturer, is not guaranteed or endorsed by the publisher.

## Footnotes

^1^elexicon.wustl.edu

^2^lsa.colorado.edu
